# Orthodontic tooth separation activates the hypothalamic area in the human brain

**DOI:** 10.1038/s41368-017-0001-y

**Published:** 2018-03-15

**Authors:** Yoshiko Ariji, Hisataka Kondo, Ken Miyazawa, Masako Tabuchi, Syuji Koyama, Yoshitaka Kise, Akifumi Togari, Shigemi Gotoh, Eiichiro Ariji

**Affiliations:** 1 Department of Oral and Maxillofacial Radiology, Aichi-Gakuin University School of Dentistry, Nagoya, Japan; 2 Department of Pharmacology, Aichi-Gakuin University School of Dentistry, Nagoya, Japan; 3 Department of Orthodontics, Aichi-Gakuin University School of Dentistry, Nagoya, Japan; 4 Brain and Mind Research Center, Nagoya University, Nagoya, Japan

## Abstract

**Objectives:**

An animal experiment clarified that insertion of an orthodontic apparatus activated the trigeminal neurons of the medulla oblongata. Orthodontic tooth movement is known to be associated with the sympathetic nervous system and controlled by the nucleus of the hypothalamus. However, the transmission of both has not been demonstrated in humans. The purpose of this study were to examine the activated cerebral areas using brain functional magnetic resonance imaging (MRI), when orthodontic tooth separators were inserted, and to confirm the possibility of the transmission route from the medulla oblongata to the hypothalamus.

**Methods:**

Two types of alternative orthodontic tooth separators (brass contact gauge and floss) were inserted into the right upper premolars of 10 healthy volunteers. Brain functional T2*-weighted images and anatomical T1-weighted images were taken.

**Results:**

The blood oxygenation level dependent (BOLD) signals following insertion of a brass contact gauge and floss significantly increased in the somatosensory association cortex and hypothalamic area.

**Conclusion:**

Our findings suggest the possibility of a transmission route from the medulla oblongata to the hypothalamus.

## Orthodontics: The nerve pathways of tooth movement

Identifying the nerve pathways involved in tooth movement could lead to better targets for pain relief. Non-steroidal anti-inflammatory drugs cannot be used to relieve orthodontic pain because they impair the processes involved in tooth movement. Yoshiko Ariji of Japan’s Aichi-Gakuin University School of Dentistry, Nagoya, and colleagues used functional MRI scans of ten healthy adult volunteers to identify the parts of the brain that become active when separators are briefly inserted between pre-molar teeth. They found separator insertion led to a significant rise in the activity of the hypothalamus and the part of the brain’s cerebral cortex associated with touch and proprioception. Together with the results of previous studies in mice, the results suggest a nerve pathway that could be targeted to alleviate pain from orthodontic procedures without negatively impacting tooth movement.

## Introduction

Tooth separation is frequently used in orthodontic tooth movement, and it usually causes uncomfortable sensations, including pain around the tooth. In animal experiments, the mechanical pressure caused by insertion of orthodontic elastics elevates neuronal activity in trigeminal spinal subnucleus caudalis (Vc) neurons of the medulla oblongata.^1–2^ Activation of Vc neurons may be transmitted to the cerebral somatosensory cortex, resulting in recognition of pain around the tooth. The activation of the somatosensory cortex by other painful stimuli has been confirmed in human beings also.^3^ Regarding tooth movement force, however, it has been confirmed solely by a recent in vivo study with rats.^4^

Meanwhile, pain as an afferent stimulus is considered to play a role in tooth movement through activation of the central nervous system.^2–5^ Tooth movements are performed by both bone resorption and formation through the sympathetic nervous system, which may be regulated by the hypothalamus.^6–7^ Indeed, the intervention of the central nervous system is verified indirectly by the fact that administration of nonsteroidal anti-inflammatory drugs (NSAIDs) for pain relief impairs the tooth movement process in orthodontic treatment.^5^ Although the activation of the medulla oblongata is verified in tooth separation.^1–2^, the route of transmission signals to the hypothalamus from the medulla oblongata has not been verified in human beings.

Although differences in afferent stimuli may cause different degrees of activation in the somatosensory cortex and hypothalamus, even activations themselves in these areas have not been confirmed in orthodontic tooth separation for human beings due to the lack of appropriate procedures. Recently, functional magnetic resonance imaging (fMRI) was introduced as an effective tool for non-invasively identifying the anatomical brain areas that are activated by afferent signals.^8–9^

Taken together, if fMRI would indicate the activation in the hypothalamus area, the possibility of a transmission pathway to the hypothalamus from the medulla oblongata could be verified. The purpose of this study was to identify the activated cerebral areas using brain fMRI with a special emphasis on the hypothalamic area together with the somatosensory cortex. In this regard, two types of orthodontic tooth separators were inserted between the upper premolars of healthy volunteers.

## Results

**Fig. 1:**
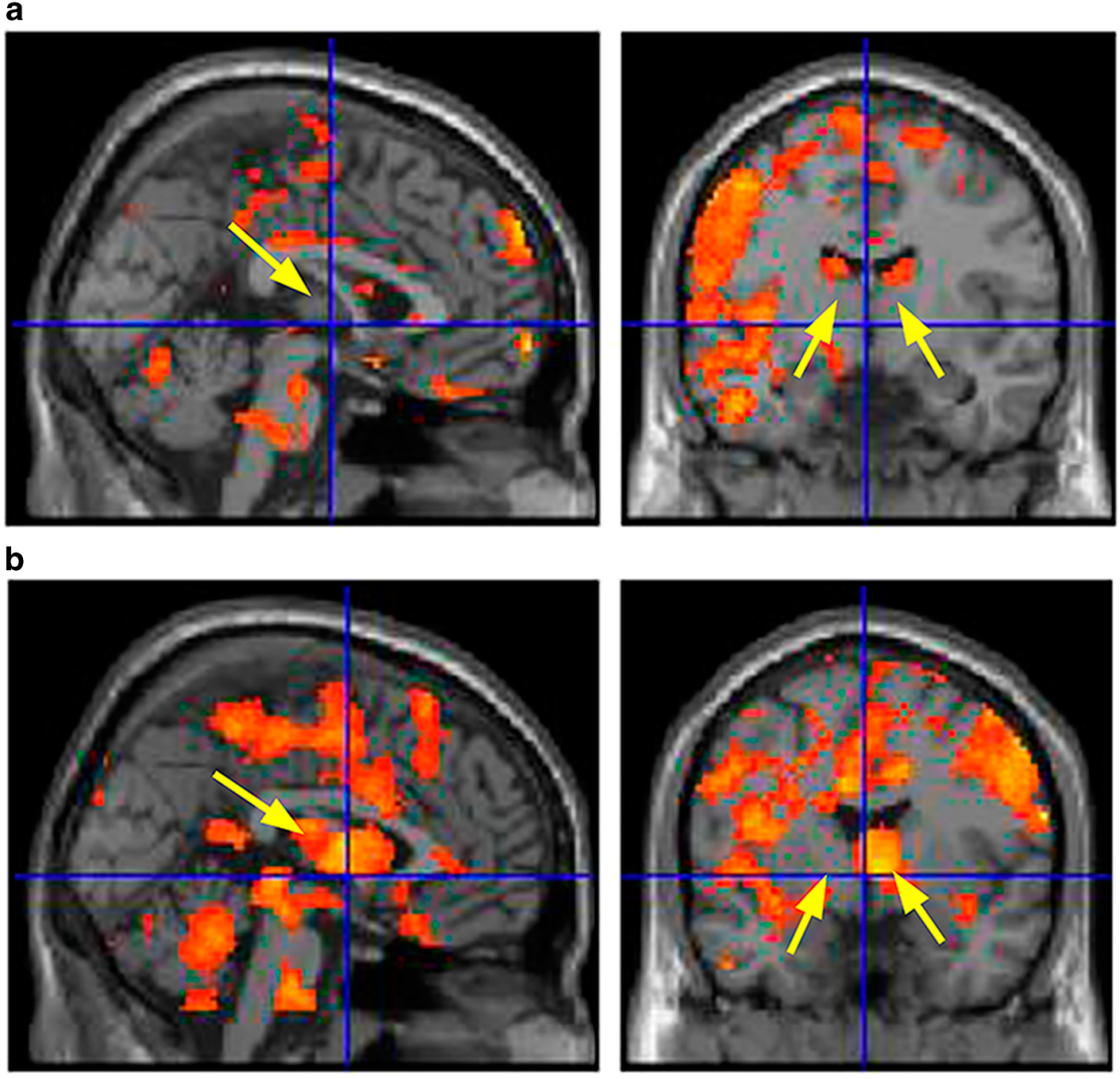
BOLD signal increases during insertion of apparatuses. **a** During insertion of floss; **b** during insertion of the brass contact gauge. Arrows show the thalamus

Table 1 shows the cerebral regions with significant increases in blood oxygenation level dependent (BOLD) signals during the insertion of the orthodontic appliances compared with the baseline (at rest). During the insertion of both appliances, BOLD signals increased in the left parietal association area, frontal association area, temporal association area, insula, and cerebellum. BOLD signals during insertion of the floss increased in these areas and in the left hippocampus and amygdala (Fig. 1a). BOLD signals during the insertion of the brass contact gauge increased in these areas and in the right thalamus, hippocampus, calcarine sulcus, left putamen, and lingual gyrus (Fig. 1b).

**Table 1:** Significant increase in fMRI signal during insertion of the apparatuses minus baseline

Region of activation	BA	Side	MNI			Maximal *t* value
			*x*	*y*	*z*	
Brass contact gauge						
Parietal association area	5	L	12	−50	20	4.02
	40	L	−26	−50	46	3.31
Frontal association area	44	L	−36	18	8	3.95
Temporal association area	20	L	−46	−50	−22	3.73
	21	L	−54	−50	20	3.42
	22	L	−48	−46	18	3.66
	37	L	−38	−62	−18	3.60
Thalamus		R	22	−24	4	3.44
Hippocampus		R	22	−8	−12	3.63
Putamen		L	−26	−16	8	3.96
Lingual gyrus		L	−10	−74	−10	4.00
Calcarine sulcus		R	10	−84	10	3.39
Insula	13	L	−40	−2	18	3.33
Cerebellum		L	−18	−56	−14	3.68
Floss						
Parietal association area	40	L	−48	−40	26	4.31
Frontal association cortex	11	L	−28	28	−8	4.90
	44	L	−46	−28	20	3.59
Temporal association area	20	L	−46	−52	−10	3.77
	21	L	−52	2	−24	3.58
	22	L	−42	−20	0	3.45
	37	L	−42	−54	−18	4.14
	38	L	−48	8	−20	3.20
Hippocampus		L	−32	−8	−22	3.31
Amygdala		L	−30	−2	−18	3.36
Insula	13	L	−34	12	−8	3.84
Cerebellum		L	−20	−46	−20	3.62

Only significant clusters of activation corrected for multiple comparisons (*P* < 0.05) were listed. The maximal *t* value indicated the most significant peak activations in each cluster.

BA, Brodmann area; fMRI, functional magnetic resonance imaging; L, left; MNI, Montreal Neurological Institute; R, right

**Fig. 2:**
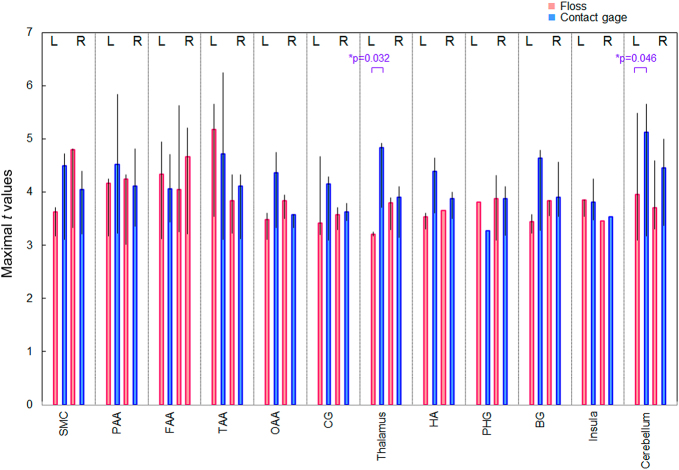
Group analysis based on individual activation sites and magnitude SMC primary somatomotor cortex (BA 1–4). BA, Brodmann area; BG, caudate nucleus putamen and pallidum; CG, cingulate gyrus; FAA, frontal association area (BA 11, 44); HA, hippocampus and amygdala; OAA, occipital association area plus cuneus and lingual gyrus; PAA, parietal association area (BA 5, 40); PHG, parahippocampal gyrus; TAA, temporal association area (BA 20–22, 37–38)

Figure 2 shows the results of group analysis based on individual activated sites and magnitude. BOLD signals were significantly higher in the left thalamus and cerebellum during the insertion of the brass contact gauge compared with the floss (*P* = 0.032 and *P* = 0.046, respectively, Wilcoxon rank sum test). In other areas, no differences were observed between them.

Visual analog scale (VAS) values of pain during the task were 51.8 ± 24.2 for the brass contact gauge and 3.3 ± 5.0 for the floss. There was a significant difference (*P* = 0.005, Wilcoxon rank sum test). VAS values of the residual discomfort after magnetic resonance (MR) examination were 24.7 ± 25.6 and 2.0 ± 2.7, respectively. There was a significant difference (*P* = 0.008, Wilcoxon rank sum test). There were no relationships between the VAS and the maximal *t*-values.

## Discussion

In the early stage of tooth separation, patients often complain of pain or discomfort.^10^ Pain itself as an afferent stimulus is considered to be related with tooth movement through the sympathetic nervous control.^2–4^ This is symbolically expressed as NSAIDs administration for the pain disturbing the tooth movement.^5^ To elucidate this mechanism, fMRI study of brain activity could provide a great contribution. The known facts are first summarized.

**Fig. 3:**
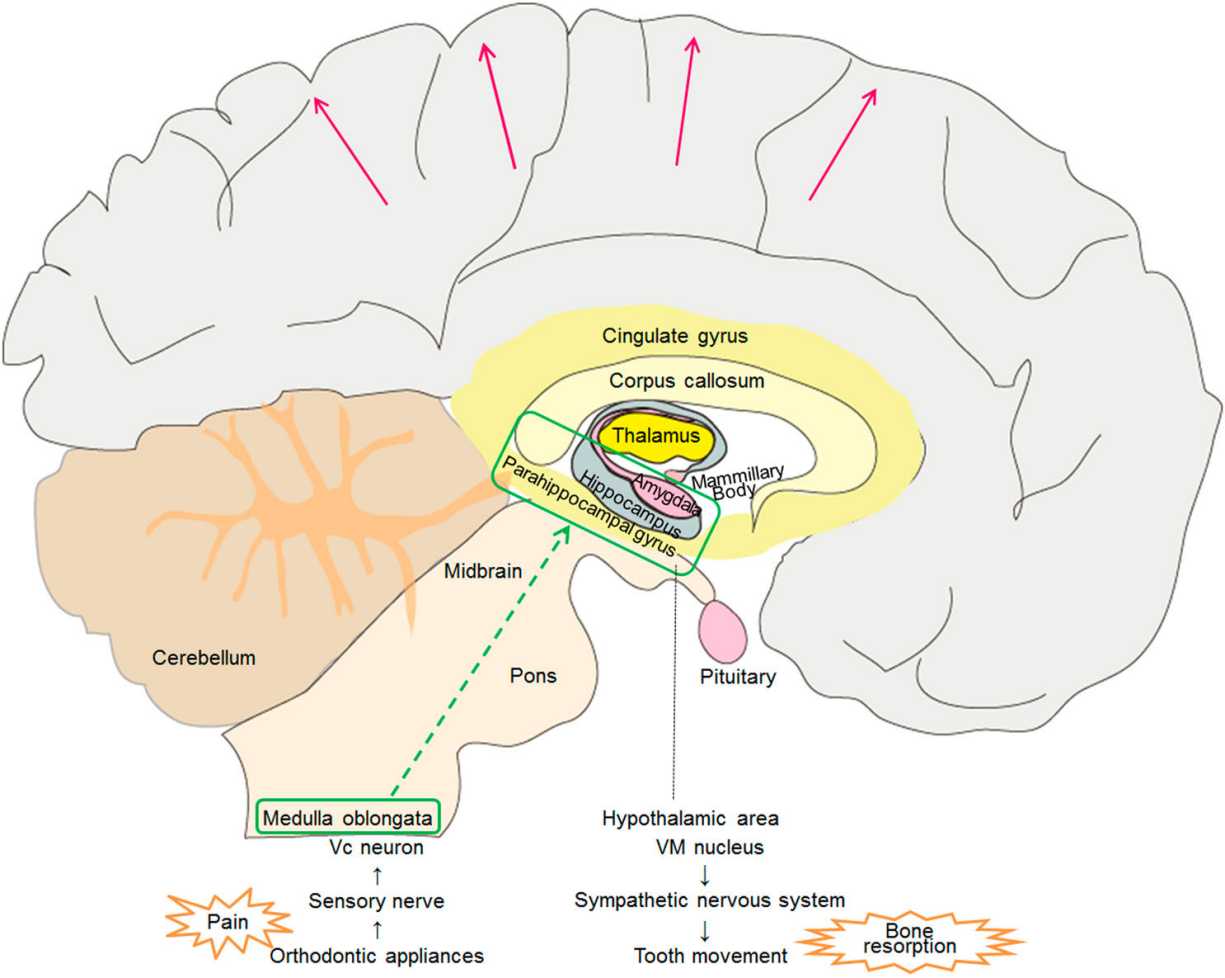
Schema of the hypothesis regarding the cerebral activated sites and transmission routes at the insertion of the orthodontic appliances. Vc, neuron trigeminal spinal subnucleus candalis neuron; VM, nucleus ventromedial nucleus

Insertion of the orthodontic appliance first activates the trigeminal spinal subnucleus caudalis (Vc) neurons of the medulla oblongata (Fig. 3). In an immunohistochemical study with rats, the mechanical pressure for tooth movement elevates the Vc neuron of the medulla oblongata.^1–2^ The stimuli activating the medulla oblongata are transmitted to the somatosensory cortex. A recent in vivo study with rats verified that the stimuli by orthodontic tooth movement could evoke the neural excitation of the cerebral somatosensory cortex.^4^ As a result, the patient feels pain or discomfort.^11^ Regarding the tooth movements, some animal experiments suggest that bone turnover is related to the sympathetic nervous system.^6–7^ In an experiment with orthodontic appliances in mice, the sympathetic neuromarkers, such as tyrosine hydroxylase, was increased in the periodontal ligament, and the number of osteoclasts was markedly increased on the bone surface.^12^ Periosteal tyrosine hydroxylase expression was decreased along with preosteoclasts and osteoclasts by experimentally destroying the sympathetic nervous system.^13–14^ Furthermore, hypothalamo-pituitary disconnection in ewes resulted in low bone turnover with depression of osteoblast and osteoclast cellular activity.^15–16^

Based on these facts, the following hypotheses were designed (Fig. 3). Appliance of the orthodontic separator would activate the Vc neurons of the medulla oblongata and the cerebral somatosensory cortex. Simultaneously, the stimuli would be transmitted from the medulla oblongata to the nucleus ventromedial nucleus (VM) neurons of the hypothalamus and could activate the sympathetic nervous system. As a result, bone resorption and tooth movement would occur. No verifications have been reported for the activation in the somatosensory cortex or the possibility of the route from the medulla oblongata to the hypothalamus in tooth separation in human beings. Although the brain fMRI could not depict the medulla oblongata, the results of this study have verified the somatosensory cortex activation and the possibility of the transmission route from the medulla oblongata to the hypothalamus. Therefore, our results provide a potential new therapeutic method, such as local administration of a sympathomimetic agent, which may allow efficient tooth movement while blocking pain.

In this experiment, two types of orthodontic tooth separators were considered: a brass wire separator and an elastomeric separator. Nalbantgil et al. investigated pain during the insertion of two types of separators in 87 healthy volunteers and determined that the brass wire caused the greatest pain and discomfort immediately after insertion, whereas the elastomeric separator caused the greatest pain during the first 2 days after insertion.^10^ The brass wire is non-magnetic and can therefore be brought into the MR room.^10^ However, the orthodontic plier, which is used for the insertion of the orthodontic separators, is magnetic and cannot be brought into the MR room. Therefore, non-magnetic alternative appliances that can be inserted by hand were used in this experiment. As an alternative to the brass wire separator, the self-made brass contact gauge was used. As an alternative to the elastomeric separator, commercially available floss was used. In this study, the floss caused only a small amount of pain, and doubt remained regarding whether the floss was a sufficient alternative to the elastomeric separator.

A possible problem during MR examination is the generation of heat by the metal. In experiments examining the effects of orthodontic appliances on radiofrequency heating during 3-T MRI, the greatest change in temperature was 3.04° by nickel-titanium arch wire and stainless steel ligature wire.^17^ Therefore, the temperature change by orthodontic appliances was considered to be within acceptable ranges clinically.^17–18^ The brass wire used in this study is non-magnetic and is expected to show smaller changes than nickel-titanium. To prevent mucosal damage by direct contact, the gauze was inserted into the buccal side of the appliance.

This experiment showed that insertion of the brass contact gauge increased the BOLD signals in the parietal association areas (somatosensory association cortex, BA 5, 40), thalamus, and hippocampus. Namely, activation of the somatosensory area associated with pain was observed, and the possibility of the transmission system including the hypothalamic area was suggested. The other activated areas (frontal association area, temporal association area, putamen, insula, and cerebellum) were associated with cognition and judgment. In contrast, the insertion of the floss also activated the parietal association area (somatosensory association cortex, BA 40) and the hypothalamic area (hippocampus, amygdala).

BOLD signals following insertion of the brass contact gauge were significantly higher in the left thalamus and cerebellum than with the floss. This result may be associated with the fact that the contact gauge caused a greater pain response during the task and the greatest discomfort after examination.

This study did not confirm whether the tooth was moved by the orthodontic separators. Davidovitch et al. reported that the insertion of the elastomeric separator for 1, 4, 12, and 24 h in 24-healthy volunteers produced a space of 0.087, 0.123, 0.184, and 0.198 mm, respectively.^19^ Furthermore, recovery to baseline after separator removal was achieved after 24 h.^19^ The amount of tooth movement by the brass wire separator was smaller than that by the elastomeric separator.^10^ With respect to these reports, an experiment involving inserting the appliances for less than 10 min during MR examination would be considered to produce a small amount of tooth movement. Recovery after appliance removal would be achieved quickly, so this experiment would result in minimal damage to the participants.

## Materials and methods

### Subjects

This study was performed with approval from the authors’ university ethics committee (No. 420). This study was planned and performed in accordance with the code of ethics of the World Medical Association (Declaration of Helsinki).

We recruited volunteers to cooperate in the research. All participants were informed of the study aims and provided consent before participating. None of the participants had any abnormalities in skeletal or occlusal status. None of the volunteers had received orthodontic treatment. Ten healthy volunteers (six men and four women; age range 26–40 years; mean age (30.5 ± 5.9) years) participated in this study.

### Task

**Fig. 4:**
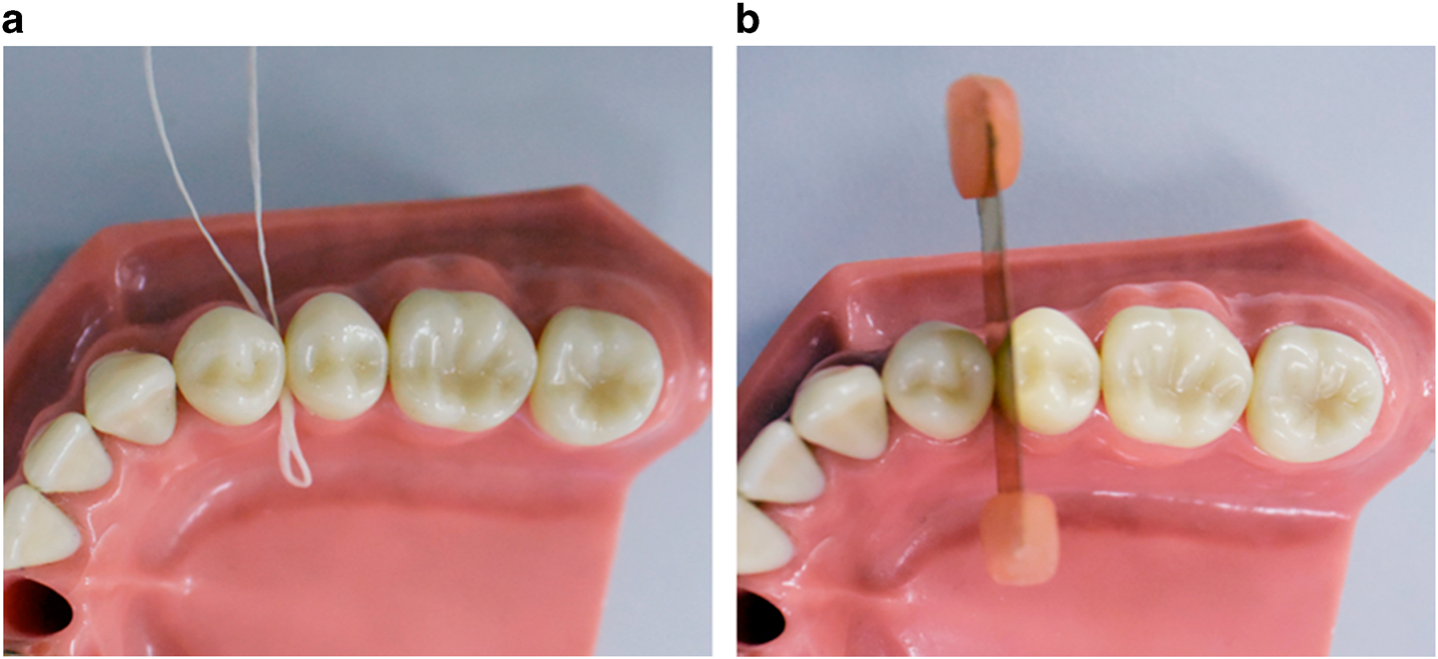
Orthodontic apparatuses used in this experiment. **a** GUM expanding dental floss with wax; **b** self-made brass contact gauge

Two types of alternative orthodontic tooth separators were inserted between the upper right first and second premolars of healthy volunteers. The orthodontic plier used to insert the orthodontic separators was magnetic and could not be brought into the MR room. Therefore, non-magnetic alternative appliances that could be inserted by hand were used in this experiment. One was commercially available floss (GUM expanding dental floss with wax, Sunstar, Inc., Tokyo, Japan), as an alternative to the elastomeric separator (Fig. 4a). Another was a self-made brass contact gauge with 0.15 mm or 0.20 mm diameter, as an alternative to the brass wire separator (Fig. 4b). The gauze was inserted into the buccal side of the apparatus to avoid touching the metal directly to the gingiva and buccal mucosa, to prevent impediments caused by heating during MRI.^14–15^

The appliances were inserted after 60 s of rest, and 30 s after the start of insertion was defined as the task. The order of insertion of the appliances was randomly determined for each subject. The different tasks were performed on separate days.

### fMRI scanning

Each participant underwent MR examination using a 3-T MAGNETOM Verio Syngo MRI scanner (Siemens, Erlangen, Germany) to allow functional T2*-weighted and anatomical T1-weighted image acquisition. Each patient’s head was fixed by tape on the forehead and a sponge on both sides of the face to prevent movement during MR examination. Whole-brain functional images were obtained using gradient echo-planar imaging sequencing, with a 3000-ms repetition time (TR); 30-ms time of echo (TE); 90° flip angle; 216-mm field of view (FOV); and 3 mm × 3 mm × 3-mm voxel resolution. Sampling consisted of 36 slices, 3 mm thick, with no gap, parallel to the anteroposterior commissure line. For each task series, 110 consecutive image volumes were acquired, including a 60-s rest, 30-s insertion (task), and 240-s recovery period.

Before functional data were acquired, anatomical images were obtained using a T1-weighted magnetization prepared rapid acquisition gradient echo (MPRAGE) sequence: (TR/TE/TI=2 500 ms/2.48 ms/900 ms, flip angle = 8°, FOV = 256 × 256 mm^2^, voxel size = 1 mm × 1 mm × 1 mm, 192 slices, no gap).

Each participant was asked to evaluate the maximal pain during the task and the degree of residual discomfort after MR examination using the VAS.

### fMRI-data processing

Preprocessing was performed with SPM 8 (http://www.fil.ion.ucl.ac.uk/spm/). The functional images were realigned to remove movement-related artefacts, normalized to the Montreal Neurological Institute (MNI) template, and spatially smoothed with a Gaussian kernel with a full width at half maximum of 8 mm.

Statistical analysis was performed with the general linear model using SPM8. For each subject, a design matrix was created using a canonical hemodynamic response function to model the response to each task. For group analysis, random effect analysis (paired *t*-test) was applied to statistically contrast images of all subjects. A value of *P *< 0.05 was considered statistically significant.

For each region of the brain, BOLD signal changes during insertion of the brass contact gauge were compared with floss (Wilcoxon rank sum test). Statistical significance was established at *P* < 0.05.

Comparisons of VAS values were performed using the Wilcoxon rank sum test. A *P* value < 0.05 was considered statistically significant.

## Conclusion

Cerebral activation following orthodontic separation was confirmed. BOLD signals at insertion of the brass contact gauge and floss significantly increased in the somatosensory association cortex and hypothalamic area. The possibility of a transmission route from the medulla oblongata to the hypothalamus was indirectly proved.
